# Transfer of Lipophilic Drugs from Nanoemulsions into Lipid-Containing Alginate Microspheres

**DOI:** 10.3390/pharmaceutics13020173

**Published:** 2021-01-28

**Authors:** Sabrina Knoke, Heike Bunjes

**Affiliations:** 1 Institut für Pharmazeutische Technologie und Biopharmazie, Technische Universität Braunschweig, Mendelssohnstraße 1, D-38106 Braunschweig, Germany; sabrina.knoke@tu-braunschweig.de; 2 Zentrum für Pharmaverfahrenstechnik (PVZ), Franz-Liszt-Straße 35a, D-38106 Braunschweig, Germany

**Keywords:** drug transfer, in vitro release, colloidal drug carriers, lipid nanoparticles, hydrogel beads

## Abstract

Knowledge about the release behavior and drug retention properties of colloidal carriers is of essential importance for quality control as well as to predict in vivo performance. When conducting release studies from such systems, the release media should preferentially contain lipophilic acceptor components in order to mimic physiological conditions. In this study, transfer from a trimyristin nanoemulsion into lipid-containing hydrogel beads was investigated for fenofibrate, cannabidiol, retinyl acetate, orlistat, and lumefantrine. To generate the acceptor system, a trimyristin nanoemulsion was incorporated into Ca-alginate microspheres (mean diameter ~40 µm) with a spraying method. Using this approach, the advantages of small lipophilic acceptor particles with a large interfacial area were combined with a single separation process from the donor via a filtration step. The method was applicable to distinguish between fast (fenofibrate) and slow drug transfer (lumefantrine) with good time resolution. Lipophilicity, estimated according to the calculated logP value of the respective drug, was a major factor influencing the transfer performance: the higher the logP value, the slower the transfer. This experimental setup is a promising technique to investigate the release of poorly water-soluble drugs from various types of nanocarriers under closer to physiological conditions than with many other methods currently applied.

## 1. Introduction

Colloidal lipid dispersions are being investigated as a promising formulation approach for the parenteral (especially i.v.) administration of poorly water-soluble drugs as they combine good bioavailability with low toxicity [1]. In order to rationally design colloidal drug carrier formulations, it is of essential importance to obtain information on their drug retention and release characteristics which can be used as quality control criteria as well as to predict in vivo behavior. Because of the small size of the carrier particles, determination of released drug is challenging. Currently, no standard regulatory in vitro release test is available for nanoparticulate systems. Several approaches can be found in the literature demonstrating the difficulties of finding a suitable method that is universally applicable.

Complete separation of the nanoparticulate drug carrier system from the released drug, as performed via ultrafiltration or centrifugation, may not always be possible. Moreover, the separation procedures may lead to artificially altered profiles of drug release because of high shear stress or long high-speed circulation times. In addition, drug release may continue during the separation process. This leads to an insufficient time resolution which may provoke distorted results [2,3,4,5]. Continuous flow methods as outlined, e.g., by D’Souza and DeLuca, are modifications of the standardized Apparatus 4 described in the United States Pharmacopoeia. A delayed response time of the instrument, fluctuations in flow rates as well as filter clogging may be difficulties associated with investigating drug release using flow-through methods [4,6].

Many authors described the use of membrane diffusion techniques (such as dialysis) which do not require a separation step [7,8]. However, rate-limiting properties that may occur with this method are widely discussed. They represent a major obstacle that needs to be considered when investigating drug release with dialysis. Depending on the method applied, erroneous results are often reported [7,9,10].

In-situ methods enable investigation of the released drug directly in the presence of nanoparticles. Unfortunately, not all drugs can be investigated using in-situ techniques since analytical drug detection methods must be available that do not interfere with the dispersed phase particles. Suitable candidates include drugs with fluorophores, electrochemically accessible groups, or acidic/basic moieties [11,12,13].

Another important consideration when investigating drug release should focus on the usage of appropriate release media. Lipophilic drugs display poor aqueous solubility. Consequently, their distribution into mainly aqueous release media, e.g., simple buffer solutions, is limited. Correlation to in vivo conditions is thus unreasonable since the physiological situation is not reflected properly. To enhance solubility in a closer to physiological way, the release medium can, for example, be supplemented with albumin [5,14]. With regard to intravenous administration, there are many lipophilic binding or distribution sites such as (lipo)proteins or cell compartments in the blood. These can act as lipophilic acceptors and should thus be present in the release media as well. Lipophilic acceptors may be added to the release media in the form of lipid particles. For example, Petersen et al. investigated the transfer of fluorescent dyes into lipophilic acceptor emulsion droplets by a flow cytometric method [15]. Hinna et al. used asymmetrical flow field-flow fractionation (AF4) to study porphyrin transfer into liposomes [16]. In some cases, the release appeared to be affected by the large size of the acceptor particles due to their limited interfacial area.

As an even closer approach to physiological conditions, the release of temoporfin from liposomes to different lipoprotein fractions in human plasma has been investigated [17,18]. Roese and Bunjes studied drug transfer into porcine serum and blood using a newly developed differential scanning calorimetry (DSC) method [19]. This way it was possible to evaluate release properties of different drugs in-situ without a separation step. As a limitation, AF4 requires a high degree of expertise as does investigating drug transfer with DSC, which additionally may be limited in applicability to donor nanoparticles with very special properties [19].

Although it is most desirable to investigate drug transfer into the physiological media relevant for administration, feasibility and applicability of many methods suffer from several drawbacks. These shortcomings are predominantly related to the small size of the nanoparticulate drug carriers on the one hand as well as the complexity of the physiological environment present in vivo on the other hand. During in vitro method development, it may be preferable to replace the very complex physiological media by simple and robust in vitro media that only contain ingredients essential for drug release.

Strasdat and Bunjes introduced a transfer setup in which a lipid nanoparticle suspension incorporated into small Calcium-alginate hydrogel microbeads served as lipophilic acceptor [20]. This method yielded promising results with the transfer of the fluorescent dye Nile red. It did, however, leave room for technical improvement and has not been applied to investigate transfer of real drugs yet. The aim of this study was to improve the microgel beads-based setup with regard to robustness and to investigate its practicability for such transfer experiments with differently loaded trimyristin donor emulsions. Fenofibrate, cannabidiol, retinyl acetate, lumefantrine, and orlistat were chosen as model drugs with different lipophilicities (estimated according to their respective calculated logP value, Table 1).

## 2. Materials and Methods

### 2.1. Materials

The triglyceride trimyristin (Dynasan^®^ 114) was donated by IOI Oleo, Witten, Germany and the surfactant poloxamer 407 (Kolliphor^®^ P127) by BASF AG, Ludwigshafen, Germany. Sodium alginate (Manugel^®^ GMB) was a kind gift from FMC International, Wallingstown, Ireland. As estimated by the supplier, the molecular weight was ~124 kDa, the content of guluronic acid (G) was 60–70% and that of mannuronic acid (M) was 30–40%. Tetrahydrofuran (HPLC grade), acetonitrile (HPLC grade), and the drugs fenofibrate and retinyl acetate were obtained from Sigma-Aldrich, Steinheim, Germany. Cannabidiol was purchased from TCH-Pharma, Frankfurt, Germany. Lumefantrine was purchased from Acros Organics, Geel, Belgium. Orlistat was donated by Formosa Laboratories Inc., Taoyuan, Taiwan. Refined rapeseed oil, sodium hydroxide, sodium azide, anhydrous glycerol, calcium chloride, acetonitrile (LC-MS grade), and tetrahydrofuran (Ultra LC-MS grade) were obtained from Carl Roth, Karlsruhe, Germany. All materials were used as received. Purified water was prepared by deionization and filtration (EASYpureTM LF, Barnstead, Dubuque, IA, USA) or was of bidistilled quality.

### 2.2. Preparation of Donor and Acceptor Lipid Nanodispersions

The nanodispersions were prepared as described previously with minor modifications [19]. The nanoemulsions consisted of 10% trimyristin as a lipid phase which was dispersed in an aqueous phase containing 5% poloxamer 407 as stabilizer. The aqueous phase was isotonized with 2.25% anhydrous glycerol and preserved with 0.05% sodium azide (*w*/*w* concentrations related to the total weight of the emulsions). Additionally, a nanoemulsion that contained 10% rapeseed oil as lipid phase was prepared. The aqueous phase of this emulsion was equal to that of the trimyristin emulsions.

The aqueous and lipid phase of the trimyristin nanoemulsions were preheated separately to 75 °C. After mixing, a pre-emulsion was formed using an Ultra-Turrax (T25 digital, IKA, Staufen, Germany) for four minutes at 11,000 rpm. Subsequently, the mixture was processed at 75 °C by high-pressure homogenization in 10 cycles at 700 bar (Microfluidizer M110-PS, interaction chamber type F12Y DIXC, Microfluidics, Newton, MA, USA) The rapeseed oil nanoemulsion was processed in the same way but at room temperature.

After homogenization, all emulsions were filtered through a polyvinylidene fluoride (PVDF) filter with 0.45 μm pore size (Rotilabo^®^, Karlsruhe, Germany) and stored in glass vials at 20 °C. Under these conditions, the trimyristin remained in a liquid state due to supercooling [19]. The trimyristin nanoemulsion served as acceptor system to be incorporated in alginate beads (cf. 2.3.). A small fraction of the trimyristin emulsion was stored at 4 °C overnight to crystallize the emulsion droplets into suspension particles which usually have a platelet-like shape [21]. The trimyristin suspension was also incorporated into hydrogel beads to be used in integrity experiments by evaluating the melting pattern via differential scanning calorimetry. Another small fraction of the emulsion was frozen (−20 °C) and, after thawing, used for integrity experiments as well. The rapeseed oil nanoemulsion was diluted with bidistilled water before being used as acceptor in transfer experiments (cf. 2.9).

For the preparation of donor emulsions to be studied in transfer experiments, fenofibrate, retinyl acetate, lumefantrine, or orlistat were dissolved in the melted trimyristin prior to emulsification. Cannabidiol was loaded passively as described previously [22]. For this purpose, the required amount of powdered drug was placed in a glass vial and trimyristin emulsion was added. The mixture was placed on a horizontal shaker (Vibrax VXR Basic, IKA-Werke GmbH & Co. KG, Staufen, Germany) and incubated at 300 rpm for about 2 days at 20 °C. After incubation, filtration of the nanoemulsion through a 0.45 µm filter (PVDF) ensured that undissolved drug was removed from the nanocarrier dispersion. All drugs were loaded at a concentration of 3% related to trimyristin (corresponding to 3 mg drug/mL emulsion). This concentration was below the solubility limit of all drugs as determined in separate studies.

### 2.3. Preparation of Trimyristin-Containing Alginate Beads

Calcium-alginate beads were produced with the spraying method as described earlier by Strasdat and Bunjes with modifications [23]. Instead of solid lipid nanoparticles, a trimyristin nanoemulsion was incorporated as acceptor into the hydrogel beads. The fluid state of the emulsion droplets provide a closer approximation to that of the lipophilic blood components (e.g., lipoproteins) [24,25]. The utilized alginate contained a high fraction of guluronic acid which is associated with an increased gel strength due to stronger cross-linking ability.

For hydrogel bead preparation, the same volume of drug-free lipid nanoemulsion and bidistilled water (approx. 20–25 mL each per batch) were mixed and 2% (*w*/*w*) sodium alginate was added to the dispersion. Under stirring at 200 rpm, the mixture was left to swell overnight. With the aid of a syringe pump (Fusion 200, Chemyx, Stafford, TX, USA), the resulting alginate-containing dispersion was fed (1 mL/min) into the two-fluid spray nozzle (diameter: 0.7 mm) of a BÜCHI Mini Spray Dryer B-191 (BÜCHI Labortechnik AG, Flawil, Switzerland) and sprayed under compressed air (650 L/h) into a continuously stirred 5% (*w*/*w*) CaCl_2_ solution (approx. 500 mL). The hydrogel particles were stored in the CaCl_2_ solution overnight to ensure thorough cross-linking. After hardening, excess CaCl_2_ solution was washed off with purified water via centrifugation (SIGMA^®^ 3–15, Sigma Laborzentrifugen GmbH, Osterode am Harz, Germany) three times at 3200 rpm. The microspheres were stored in water and used as acceptor particles. The resulting volume of each batch of dispersion was about 40–50 mL in total, of which approximately 35–50% was free water surrounding the hydrogel beads. The overall lipid concentration of each batch was evaluated via DSC (cf. 2.7.).

### 2.4. Particle Size Analysis

The particle size of the lipid nanodispersions was measured by photon correlation spectroscopy (PCS) using a Zetasizer Nano ZS (Malvern Instruments, Worcestershire, UK). Prior to the measurement, samples were diluted with purified water to an appropriate scattering intensity (attenuator 5–6). Following an equilibration time of 300 s, three consecutive measurements of 5 min each were performed at 25 °C using a laser wavelength of 633 nm at an angle of 173°. The intensity weighted mean diameter (z-Average) and the polydispersity index (PdI) were calculated as average out of three runs.

The particle sizes of the microgel beads were determined with laser diffraction (LD; Beckman Coulter LS 13 320, Beckman Coulter GmbH, Krefeld, Germany). In the measuring chamber, the samples were diluted with water to an appropriate optical density. Three consecutive measurements of 90 s each were averaged and the volume distribution, mean particle size, and D_10_, D_50_, and D_90_ values were calculated using Fraunhofer approximation.

### 2.5. pH Measurements

The pH measurements were carried out with a FiveEasy pH meter with an InLab Semi-Micro electrode (Mettler Toledo, Gießen, Germany). A 2-point calibration was performed at pH 4.01 and 9.21 in advance of each measurement series.

### 2.6. Microscopy

In order to characterize the microscopic appearance of the hydrogel beads, the samples were placed on a microscope slide and investigated under a Leica DMLM microscope (Leica Microsystems GmbH, Wetzlar, Germany) up to a 500-fold magnification. Digital photographs were taken with a Leica MC 170 HD microscope digital camera (Leica Microsystems GmbH, Wetzlar, Germany).

### 2.7. Differential Scanning Calorimetry

Differential scanning calorimetry (DSC) measurements were carried out with a DSC 1 calorimeter (Mettler Toledo, Gießen, Germany) equipped with an FRS 5 sensor that was calibrated with indium. The calibration was checked by measuring indium before a series of measurements. About 20 mg of the samples were accurately weighed into 40 µL aluminum pans (Mettler Toledo, Gießen, Germany) which were hermetically sealed by cold welding. An empty pan was used as reference and all measurements were carried out under nitrogen purge.

To examine the trimyristin concentration in the Ca-alginate microbead dispersion as well as in the drug-loaded and unloaded trimyristin emulsions, samples were heated from 20 °C to 70 °C (20 K/min) and then cooled to −5 °C with a scan rate of 10 K/min. The crystallization enthalpies obtained from the cooling curves were evaluated and the lipid content was calculated using a calibration curve obtained from measuring different amounts of bulk trimyristin.

To verify the integrity of the enclosed nanoemulsion, samples were heated from 20 °C to 70 °C (2 K/min or 20 K/min), cooled to −5 °C (10 K/min), and heated to 70 °C (2 K/min) a second time. The melting patterns of the heating curves were examined to characterize the structure of the incorporated lipid particles in comparison to those of unencapsulated counterparts.

The onset value of the crystallization signal was determined as indicator for the crystallization temperature (T_cryst_). Orlistat-containing nanoemulsions were cooled from 25 °C to 0 °C with a scan rate of 2.5 K/min. The changes in T_cryst_ of orlistat-loaded nanoemulsions were used to quantify orlistat as described in earlier studies [19]; cf. 2.9.

### 2.8. Lipid Quantification Via High Performance Liquid Chromatography

The trimyristin content of unloaded nanoemulsions was quantified with a Dionex UltiMate 3000 high performance liquid chromatography (HPLC) system equipped with a LPG-3400SD pump, a WPS-3000TSL autosampler, and a Corona Veo Charged Aerosol detector (Thermo Fisher Scientific, Waltham, MA, USA). The column (Thermo Fisher Scientific Hypersil Gold C18, 2.1 × 150 mm^2^, 1.9 μm) was kept at 25 °C and the flow rate was set to 0.3 mL/min. The mobile phase consisted of acetonitrile/tetrahydrofuran 70/30 (*v*/*v*) for trimyristin.

Samples were diluted in tetrahydrofuran/acetonitrile 50/50 (*v*/*v*) to an appropriate response; 1 μL was injected and detected at a nebulizer temperature of 50 °C. Every sample was diluted twice and every dilution measured two times (*n* = 4). Trimyristin amounts were calculated with the Chromeleon 7.2 software (Thermo Fisher Scientific, Waltham, MA, USA) using a calibration curve of trimyristin in different concentrations.

### 2.9. Investigation of Drug Transfer

Drug-loaded nanoemulsions were mixed with water-diluted Ca-alginate beads in 3 mL glass vials in a donor (d) to acceptor (a) lipid mass ratio of 1 + 9 (based on the results of lipid determination by DSC). This lipid ratio was chosen to ensure comparability of the present transfer results with those of a previous study that was performed with a DSC method [19]. The acceptor lipid content of approx. 45 mg/mL allowed samples to be taken from the donor system at predetermined time points by filtration [20]. For this purpose, a polyethersulfone (PES) membrane with 1.2 μm pore size (Pieper Filter GmbH, Bad Zwischenahn, Germany) was mounted into a custom-built screw cap that was attached to the transfer vial 30 s before sampling.

The transfer started when the required amount of donor emulsion (approx. 70–100 µL) was added to the acceptor particle dispersion (approx. 1200–1700 µL). During transfer, the samples were placed on a horizontal shaker (Vibrax VXR Basic, IKA-Werke GmbH & Co. KG, Staufen, Germany) and agitated with 300 rpm at 23 °C. For each time point of sampling, a separate transfer vial was used. The vial was turned upside down and the sample was taken by withdrawing the aqueous donor system through the membrane into a 2 mL plastic syringe (B. Braun Deutschland GmbH & Co. KG, Melsungen, Germany).

Drug load of the nanoemulsions as well as the remaining amount of drug in the nanoemulsions during transfer experiments was quantified via UV spectroscopy (Specord 40, Analytik Jena AG, Jena, Germany). Samples were dissolved in tetrahydrofuran/water 90/10 (*v*/*v*) and measured at wavelengths of 212 nm (cannabidiol), 287 nm (fenofibrate), 290 nm (lumefantrine), or 360 nm (retinyl acetate) three times. Where required, the measured absorptions were corrected for the blank absorptions of the dissolved unloaded nanoemulsion that had been treated in the same way as the respective drug-containing nanoemulsion. To ensure linearity, calibration curves for each drug were obtained by preparing at least five different dilutions containing varying amounts of the respective drug. The amount of transferred drug was calculated by subtracting the amount in the sampled aqueous donor system from the originally applied one.

Orlistat could not be quantified via UV spectroscopy in the presence of trimyristin due to the absence of an appropriate chromophore. Thus, orlistat transfer from the trimyristin nanoemulsion into the lipophilic acceptor was investigated by DSC. The change in crystallization temperature (ΔT_cryst_, determined upon cooling) is in a linear relation to the decrease in drug content [19]. An unloaded trimyristin nanoemulsion with comparable characteristics (which corresponded to 0% drug transfer; measured as control), diluted with the acceptor system in the same ratio, and the respective donor emulsion, also diluted with the same volume of the acceptor system but without lipid (which corresponded to 100% drug transfer), were used to calculate the transferred amount of orlistat by applying the rule of three.

In order to investigate the transfer of lumefantrine at a higher pH, a fraction of the donor emulsion as well as the utilized gel particle dispersion were adjusted to pH 10.8 using NaOH (1 M). Particle sizes were additionally checked directly after pH adjustment and again after the end of the respective experiment (550 h).

The filter membrane was tested regarding material stability and drug adsorption. For this purpose, three different control samples containing varying volumes of each drug-loaded lipid nanoemulsion diluted with water were prepared. The drug content in these samples was analyzed before and after filtration through the PES membrane.

Additionally, a rapeseed oil nanoemulsion was used as acceptor to investigate the transfer of orlistat with a barrier-free method according to previous studies performed by Roese and Bunjes [19]. The same lipid mass ratio of 1 + 9 between donor and acceptor was applied. The rapeseed oil nanoemulsion was diluted with water in order to attain a comparable overall lipid concentration as with the hydrogel beads-based setup (approx. 45 mg/mL lipid). Fifty microliters of the donor emulsion were added to the water-diluted acceptor emulsion (950 µL). Samples were withdrawn and directly measured via DSC as described above to quantify orlistat. In contrast to the hydrogel beads-based setup, no filtration step was necessary using this method. The presence of rapeseed oil did not influence the crystallization temperature (T_cryst_) of the trimyristin emulsion within the time frame of the experiment as confirmed in a control experiment.

In the following, all transfer results are presented by plotting the fraction of transferred drug (%) against the time (hours, h). All transfer studies were performed in triplicate.

## 3. Results

### 3.1. Characteristics of Donor and Acceptor Particles

#### 3.1.1. Particle Sizes

**Figure 1:**
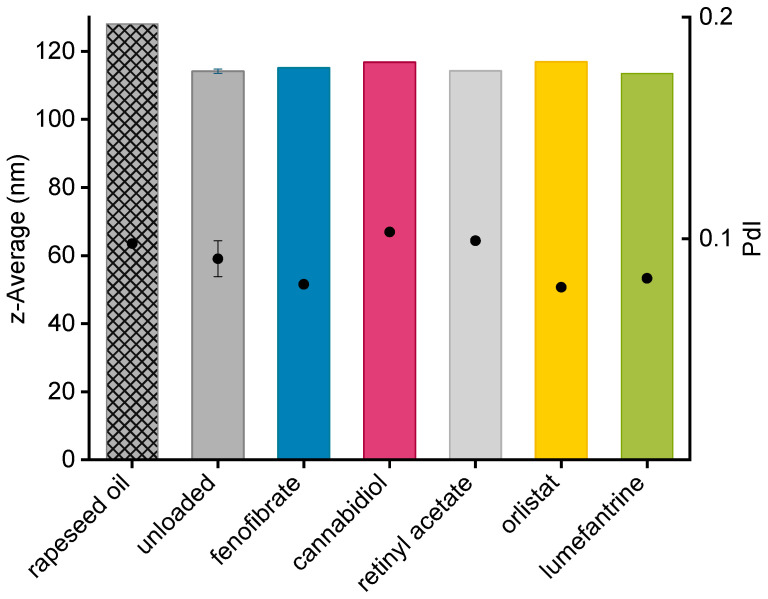
Droplet size (z-Average, represented as bars) and polydispersity index (PdI) values (PdI, represented as dots) of different batches of unloaded trimyristin emulsions (*n* = 6 ± standard deviation), drug-loaded donor emulsions (also with trimyristin matrix) as well as the rapeseed oil emulsion (patterned bar) as determined via photon correlation spectroscopy.

The particle sizes of the loaded and unloaded lipid nanodispersions were measured by PCS within a few days after production. All z-Average diameters of trimyristin dispersions were between 110 and 120 nm with PdIs between 0.08 and 0.11 indicating a monomodal size distribution (Figure 1). Storage at 4 °C caused a transformation of the supercooled lipid droplets into crystalline particles (as confirmed by DSC measurements), which differed in size by only 2 nm. If a donor emulsion was not utilized directly after production, the particle size of the emulsion was checked again after the end of the respective transfer experiment to ensure a stable system within the time frame of the investigation (maximum variations of 2 nm were observed). The z-Average of the rapeseed oil nanoemulsion was 128 nm with a corresponding PdI of 0.10.

The sizes of each batch of hydrogel particles, produced with the spraying method, were determined via LD (Table 2).

**Figure 2:**
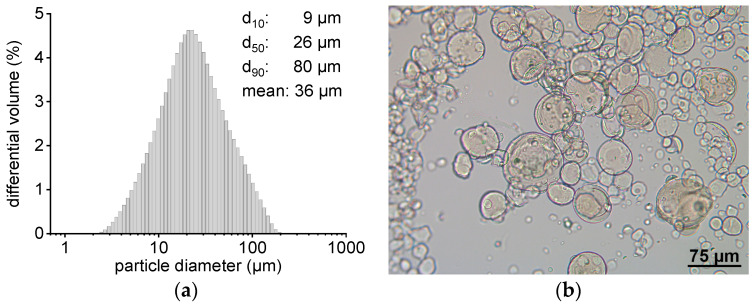
(**a**) Particle size distribution (determined via LD) of an exemplary microsphere batch that was used for fenofibrate transfer. (**b**) Light microscopic image of the same microbeads batch.

Previous investigations revealed a significantly slower drug transfer when larger gel particles were used in comparison to smaller ones. This is probably related to the presence of a higher diffusion barrier [20]. In order to minimize the diffusion barrier during the transfer experiments performed here, hydrogel particles with a mean diameter of 36–42 µm were produced and utilized in the transfer experiments. The D_10_ diameter was not below 9 µm so that the acceptor gel particles could be separated completely from the nanosized donor via filtration. It was possible to produce microgel particles with a good reproducibility (Table 2). The volume size distribution of an exemplary batch of hydrogel beads (Figure 2a) indicates a rather broad particle size distribution. This heterogeneity was, however, considered to be acceptable with regard to the aim of this study. Figure 2b shows a microscopic image of the same batch of exemplary microgel particles displaying their predominantly spherical shape.

#### 3.1.2. Drug Load of Donor Emulsions

**Figure 3:**
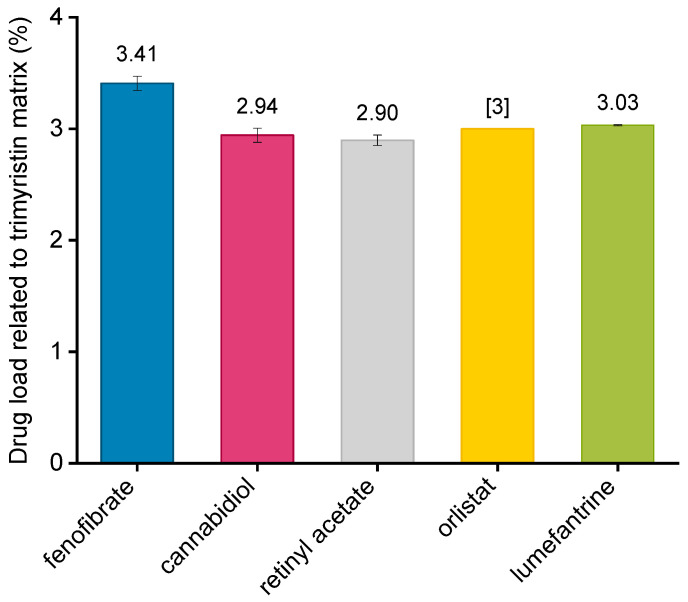
Drug loads of emulsions in relation to trimyristin as determined via UV/VIS spectroscopy. The drug load of orlistat was not quantified and the respective value [digit] is the weighed-in amount. Each value represents mean ± standard deviation (*n* = 3).

Depending on the drug, loading into nanoemulsions was performed either by dissolving the drug in the melted trimyristin prior to homogenization (fenofibrate, orlistat, retinyl acetate, lumefantrine) or after preparation via passive loading (cannabidiol). The respective drug loads are shown in Figure 3. All drugs were loaded in a concentration of ~3% in relation to the trimyristin matrix. It should be kept in mind that the given concentration of orlistat is not the actual value since it was not possible to determine orlistat by UV spectroscopy due to an insufficient light absorbance of the drug in presence of trimyristin. This does not affect the accuracy of the calculated amount of transferred orlistat as explained in Section 2.9. None of the drugs displayed significant adsorption to the PES filter membrane since recovery after filtration was close to 100% for all drugs (data not shown).

#### 3.1.3. Lipid Concentration and Nanoemulsion Integrity in Hydrogel Particles

Knowledge of the lipid content in the acceptor hydrogel microparticles is important for the adjustment of the lipid mass ratio between donor and acceptor in the transfer studies. DSC measurements were carried out to evaluate the trimyristin content in the microparticles, loaded, and unloaded emulsions, whereas HPLC measurements were performed for the same unloaded emulsions to compare and verify the lipid amount calculated from the crystallization enthalpies obtained with DSC. This was done because dispersed triglycerides are prone to forming different polymorphs that exhibit different crystallization enthalpies which may falsify the lipid quantification via DSC [21].

**Figure 4:**
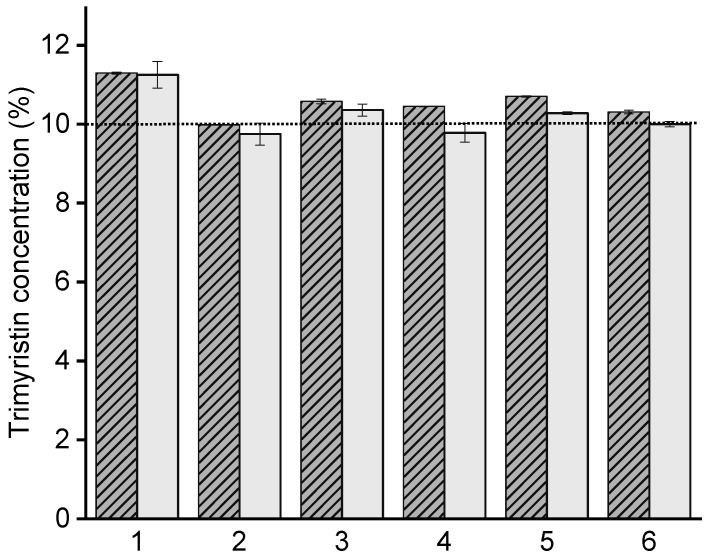
Comparison of trimyristin quantification of six different unloaded nanoemulsions via DSC (patterned bars; *n* = 3 ± standard deviation) and HPLC (uniform bars; *n* = 4 ± standard deviation). The dotted line represents the weighed-in amount.

As presented in Figure 4, the lipid content obtained by HPLC was slightly smaller for some unloaded emulsions than by evaluating the crystallization signal, but the difference was within a reasonable range. A reason for the difference might be the higher standard deviation related to the HPLC detection. The reproducibility of the DSC method was much higher. Since it was not possible to completely dissolve the microparticles in an appropriate solvent such as tetrahydrofuran or acetonitrile to extract the lipid and perform HPLC measurements, the lipid contents in the microparticles as well as in the donor emulsions were evaluated with the DSC method and used to control the lipid mass ratio in transfer experiments. A concentration of >10% trimyristin was most likely due to excessive water evaporation because of long processing times during emulsion production in the heat.

In general, the lipid content of the microparticle dispersions were adjusted to ~45 mg/mL trimyristin by adding water. A certain fraction of water surrounding the microparticles was required to assure thorough mixing of donor and acceptor on the one hand and to be able to draw a sufficient volume of sample out of the transfer vial for drug quantification on the other hand. The lipid concentration of the microparticle dispersions used for transfer studies as determined via DSC is stated in Table 2.

**Figure 5:**
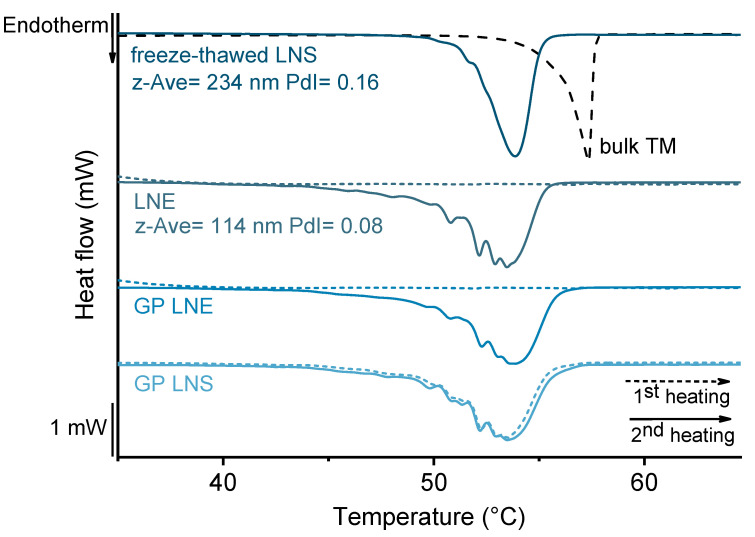
DSC heating curves of the original lipid nanoemulsion (LNE), lipid nanoemulsion and lipid nanosuspension incorporated into hydrogel particles (GP LNE and GP LNS, respectively) and a lipid nanosuspension measured after a freeze-thaw cycle (freeze-thawed LNS). Comparison of the signals of the first (dotted lines) and the second heating run (solid lines); the dashed line displays the melting signal of bulk trimyristin (TM).

Preserving the properties of the incorporated lipid nanoemulsion, especially its particle size distribution, was an important aim for the use of lipid-filled hydrogel microparticles as acceptor in the transfer experiments. During spraying upon microgel production, the nanoemulsion-containing alginate dispersion was exposed to high shear forces which may have a negative effect on the integrity of the enclosed nanoemulsion. DSC was used to characterize the structure of the enclosed lipid nanoparticles via investigation of the melting pattern [23]. For the integrity experiments performed here, the same emulsion batch was used for all experiments (Figure 5). In the case of the lipid nanoemulsion (LNE) and the (same) lipid nanoemulsion enclosed in gel particles (GP LNE), no melting event was observed during the first heating run. This confirmed that the trimyristin droplets remained in a supercooled liquid state. After the cooling step that induced crystallization of the emulsion droplets, encapsulated nanoemulsion as well as untreated emulsion revealed a characteristically structured melting pattern indicating the presence of small, platelet-shaped nanoparticles [21].

In contrast, the signals of the emulsion whose particle size distribution had been deliberately damaged by freezing (freeze-thawed LNS) and that of the trimyristin bulk material (bulk TM) were unstructured and appeared at higher temperatures. Yet, the melting pattern of the encapsulated nanoemulsion differed slightly from that of the untreated emulsion: the signals were less sharp and were shifted minimally to higher temperatures. This phenomenon was, however, also observed when the lipid nanosuspension (formed from the same nanoemulsion by storage <4 °C) was directly incorporated into microgel particles (GP LNS). A comparison of the melting patterns obtained during the first and the second heating run of the suspension inside the hydrogel beads indicates, that this disparity does not originate from the encapsulation process itself but from the presence of the alginate matrix during crystallization of the nanoemulsion. Heating of the nanoemulsion enclosed in microbeads for the second time thus does not display the melting pattern of the original particle size distribution as it was after encapsulation but an artefact due to the measurement procedure. An explanation for this was discussed earlier [23,26,27]; the phenomenon may originate from reorganization of emulsifier molecules during heating and cooling in the DSC. In consequence of these findings, which are in accordance with those of Strasdat and Bunjes, it is assumed that supercooled lipid emulsion droplets still existed in the gel particles and retained a very similar size distribution after encapsulation.

### 3.2. Investigation of Drug Transfer

**Figure 6:**
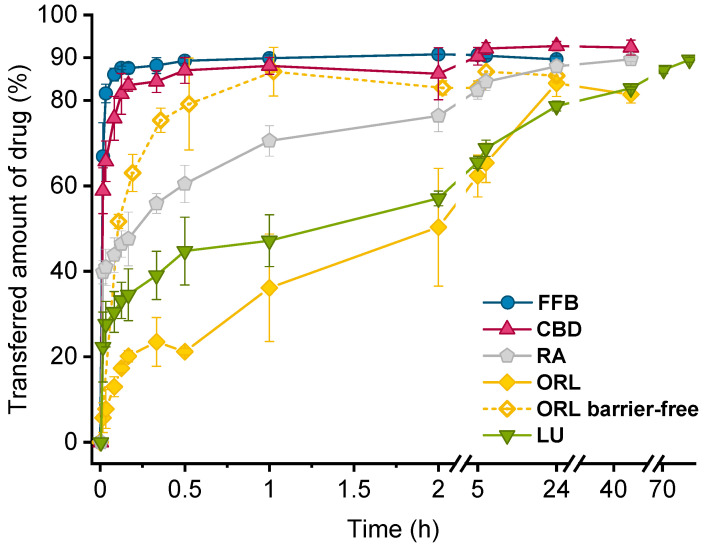
Drug transfer into trimyristin nanoemulsion-containing microgel beads or a rapeseed oil nanoemulsion (barrier-free; dotted line). Each value represents mean ± standard deviation (*n* = 3). Abbreviations: FFB = fenofibrate, CBD = cannabidiol, RA = retinyl acetate, ORL = orlistat, LU = lumefantrine.

Drug transfer from trimyristin emulsions (d) into trimyristin-containing alginate microspheres (a) was investigated for five different drugs loaded in a concentration of ~3% (drug related to lipid matrix). A lipid mass ratio of 1 + 9 (d + a) was used in all studies. After mixing of donor and acceptor, fenofibrate transferred very quickly and a plateau was reached within a few minutes (Figure 6). The achievable time resolution of the hydrogel beads-based method was higher than in other experimental setups [2,28]. It was possible to draw the first sample within 60 s after homogenous mixing of donor emulsion and acceptor inside the transfer vial. The plateau for fenofibrate transfer was at about 90%. This confirmed the assumption of an equal distribution of fenofibrate between the trimyristin matrices of donor and acceptor particles based on the adjusted lipid mass ratio. In earlier studies [20], the expected plateau value was not reached, possibly due to water loss from the gel particles during the sampling process. Such an effect was not observed in the present study. Obviously, compared to the previous study, the stability of the gel particles could be improved by using a sodium alginate quality with a higher G:M ratio and by utilizing a higher alginate concentration (2% vs. 1%).

The course of the transfer was very similar to that observed for fenofibrate with an in-situ method in previous studies: Roese and Bunjes investigated the transfer into a rapeseed oil nanoemulsion without any distorting effects of diffusion barriers and monitored the process in real-time [19]. Strasdat and Bunjes observed considerably lower transfer rates for Nile red when the acceptor suspension was incorporated in hydrogel beads with sizes D_50_ > 350 µm [20]. Based on findings for transfer into smaller hydrogel beads (D_50_ < 50 µm), the authors concluded that the diffusion barrier only seemed to be experimentally relevant for significantly larger gel particles. These findings are in accordance with the experiment with fenofibrate in the current study. It seems that a diffusion barrier did not hinder the transfer of fenofibrate in a distinct manner. Otherwise, the transfer course of fenofibrate should have deviated more significantly from that of the barrier-free in-situ method.

The transfer of cannabidiol was also completed after a very short period of time and a plateau of approximately 90% transfer was reached. All other drugs exhibited a slower transfer. After one hour, about 70% of retinyl acetate, ~47% of lumefantrine, and ~36% of orlistat had transferred from the donor into the acceptor compartment (Figure 6 and Table 2). The expected plateau value of approximately 90% was reached after about 40 h for retinyl acetate and >70 h for lumefantrine. Transfer of orlistat was detected from the change in crystallization temperature of the trimyristin nanodroplets. The difference between loaded and unloaded emulsion was small (0.51 °C for 3% orlistat). As a consequence, minor fluctuations in T_cryst._ caused high deviations. Orlistat transfer might thus not have been completed after the end of this experiment (41 h).

The orlistat transfer observed with the hydrogel beads-based setup was compared with a transfer in a barrier-free scenario. For this purpose, the DSC method according to [19] was applied using a rapeseed oil nanoemulsion as acceptor. It has been reported that dilution of donor and acceptor leads to a decrease in transfer rates [15]. With that in mind, the same dilution of donor and acceptor that was used during the transfer with hydrogel particles was applied in this barrier-free study by adjusting the overall lipid concentration (without changing the d + a ratio). In the barrier-free setup, transfer of orlistat was significantly faster and was completed after about 1 h (Figure 6, dotted yellow line).

Drug transfer between lipophilic particles may occur via collision or by diffusion through the aqueous phase [29]. Since, in the hydrogel beads-based setup, the acceptor emulsion is immobilized in the hydrogel matrix, collision driven transfer is not assumed to play a significant role. Instead, diffusion is likely to be the predominant transfer mechanism. The differences in orlistat transfer observed with the hydrogel beads-based setup and the barrier-free method thus probably originate from the presence of a diffusion barrier provided by the alginate matrix. Additionally, the donor droplets were dispersed only in the water surrounding the hydrogel particles whereas, in the barrier-free setup, donor and acceptor nanoemulsion droplets were not kept in different compartments but were homogenously mixed. In consequence, the diffusion paths for the drug molecules in the hydrogel beads-based setup were much longer in comparison to those in the barrier-free method. This might be a reason for the slower transfer of orlistat observed with the hydrogel particle-based setup.

These effects appeared to be much more critical for highly lipophilic substances since fenofibrate and cannabidiol (both less lipophilic according to their calculated logP value) exhibited a faster transfer into the lipid-containing hydrogel beads. Partition into the water phase is considerably higher for drugs with a low calculated logP value. Consequently, the concentration gradient between the water phase surrounding the hydrogel beads and the acceptor nanoemulsion within the alginate matrix was higher for drugs with a lower logP value, resulting in a faster transfer rate. At this point of investigation, however, a final conclusion on the cause for the observed differences in orlistat transfer rate is difficult since several parameters may influence the underlying mechanisms. Considering the contributing factors described above, it might be possible that an over-discriminatory effect was observed for the transfer of orlistat (and consequently other very lipophilic drugs under investigation) with the hydrogel beads-based method.

A major advantage of the microbeads-based method is its suitability for the investigation of a wide range of drugs as well as carrier systems. Unlike with many other methods, no specific analytical properties of the drug or experimental setup e.g., fluorescent or electrochemically active drugs [11,13,15] or the use of a special carrier material [19] are required.

**Figure 7:**
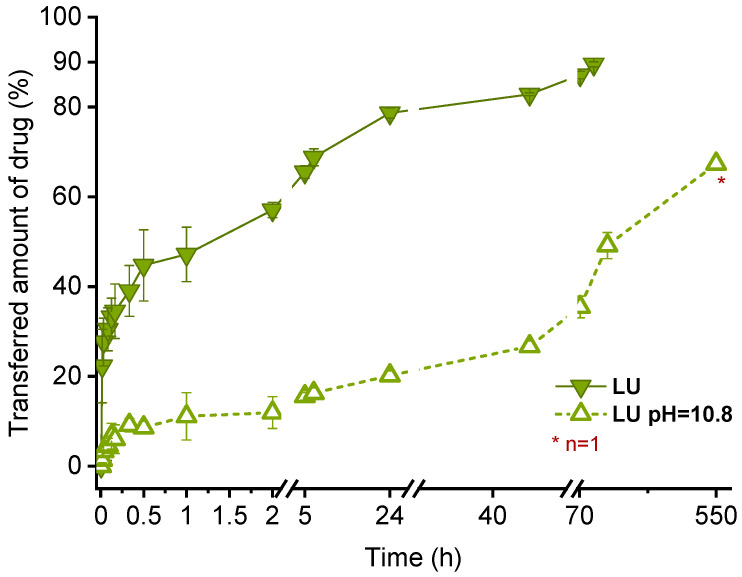
Comparison of lumefantrine transfer before (same data as in Figure 6 but different abscissa scaling) and after pH adjustment. Each value represents mean ± standard deviation (*n* = 3).

Comparing the transfer results of this study, the fastest transfer was observed for fenofibrate, which was chosen as drug with the lowest lipophilicity (logP 4.86), and conversely for orlistat (logP 7.61), appearing to be the most lipophilic drug in this experimental setup. However, the logP values presented in Table 1 suggest lumefantrine as the substance with the highest lipophilicity (logP 8.34). In neutral to slightly basic dispersions, lumefantrine would be expected to be ionized to some degree (pKa 8.71; obtained from Scifinder, calculated by ACD/Labs Software V11.02 at 25 °C). No control over pH was employed for the acceptor dispersion, so it can only be assumed that the pH was around 6.5 during transfer experiments. This was the pH measured in a different microparticle dispersion, produced and handled the same way as the microparticle dispersion which was used for investigations of lumefantrine transfer. The pH of the lumefantrine donor emulsion was approximately 6.9 (measured after the transfer experiments were performed). In this case, not the logP but the logD value (predicted to be 6.17 at pH 6 and 7.04 at pH 7, respectively) had to be considered to evaluate the lumefantrine transfer in relation to the other drugs. Setting the pH to 10.8 in the lumefantrine donor emulsion as well as the respective acceptor dispersion should render lumefantrine uncharged, leading to a slower transfer due to higher lipophilicity. This assumption was confirmed by measuring lumefantrine transfer at this pH, being only about ~10% completed after one hour (Figure 7). Even after one week (168 h) the transfer continued and was not complete at the end of the experiment (67% drug transfer after 550 h).

The change in pH neither affected the size of the donor emulsion nor of the microgel particles as confirmed by particle size measurements. Thus, this method appeared to be applicable for prolonged transfer studies, even at high pH values. All other drugs examined in this study did not have acidic or basic moieties and would thus not have been affected by any ionization at different pH values.

In agreement with previous studies [15,19,30,31,32], lipophilicity, estimated based on the calculated logP values of the drugs (logD for LU, respectively), was a major factor determining the course of the drug transfer. This indicates that the transfer observed in these experiments is under distribution control and that the characteristics of the drug (and not those of the carrier system) dominate the release performance. Takino et al. postulated that a logP value larger than 9 was required to accomplish sustained drug release from lipid nanoemulsion droplets [33]. Their conclusion is congruent with the findings of this study.

Using the trimyristin nanoemulsion enclosed in hydrogel beads as a lipophilic acceptor system is a promising approach to mimic the lipophilic compounds present in the bloodstream. Other studies analyzed the transfer properties of the model drug temoporfin from liposomes to the individual lipoprotein fractions and albumin in human plasma. Significant differences regarding the distribution profiles of the drug into the different lipophilic acceptors were found [17,18,34]. Concerning the relative abundance of the different (lipo)protein fractions in human plasma, the highest proportion can be attributed to albumin (~55%) [35]. In other studies, the addition of albumin enhanced the solubility of the drugs compared to purely aqueous release media [5,14]. Trimyristin alone may thus not be sufficiently representative as ingredient of “model-blood” since other lipophilic components such as phospholipids and (lipo)proteins might have an impact on drug distribution as well.

Therefore, modifications of the enclosed lipophilic system, e.g., by incorporating cholesteryl esters or albumin, might offer an even more realistic approach to the variety of relevant lipophilic acceptors in the blood which might be investigated in further studies.

## 4. Conclusions

The trimyristin-containing hydrogel particles could be applied successfully to investigate the transfer of drugs with different lipophilicities from lipid nanoemulsions. The method was applicable to distinguish between very fast and slower drug transfer with good time resolution by combining the advantages of small acceptor particles (and thus large specific surface area) with a simple separation procedure from the donor particles by filtration. The enclosure of trimyristin nanodroplets was a simple approach to mimic lipophilic compounds present in the bloodstream and thus created an experimental setup which is closer to physiological (i.v.) conditions than with most other release media currently applied.

The short time to achieve the plateau value of transfer for fenofibrate and cannabidiol justifies the conclusion that this donor system is a burst release vehicle and that the prolonged transfer observed for retinyl acetate, lumefantrine, and orlistat is predominantly determined by their lipophilicity (estimated according to their calculated logP value) and thus is partition driven.

A diffusion barrier appeared not to be experimentally relevant for fenofibrate and cannabidiol that exhibited a fast transfer but seemed to be more critical for rather slowly transferring drugs like orlistat. The hydrogel beads-based setup offers the possibility to investigate a broad variety of colloidal carriers (e.g., nanoparticles based on lipophilic polymers like PLGA, liposomes or liquid crystalline nanoparticles) with regard to drug retention and controlled release properties. Testing the release behavior of many different moderately lipophilic drugs with this method appears to be widely applicable since detection is not limited to specific analytical characteristics. However, the transfer course of drugs with very high logP values obtained with this method should be interpreted with appropriate care and possible rate-limiting aspects have to be considered. Whether this would also apply for colloidal carriers with pronounced controlled-release properties that release only very limited amounts of drug per unit time remains to be investigated.

## Figures and Tables

**Table 1:** Calculated octanol-water partition and distribution coefficients (LogP and LogD) of the drugs under investigation. (Source; calculated by).

Drug	Abbreviation	LogP (Drugbank; ALOGPS)	LogD (SciFinder; ACD/Labs)
Fenofibrate	FFB	4.86	-
Cannabidiol	CBD	6.1	-
Retinyl acetate	RA	6.56	-
Orlistat	ORL	7.61	
Lumefantrine	LU	8.34	6.17 (pH 6) 7.04 (pH 7)

**Table 2:** Size and lipid content of the different batches of lipid-containing hydrogel microbeads and transfer results obtained applying the respective hydrogel beads. All values *n* = 3 batches ± standard deviation or *n* = 2 batches (marked with *).

Investigated Drug	Mean Diameter (µm) ± SD	D_10_ (µm) ± SD	D_90_ (µm) ± SD	Lipid Content (mg/mL) ± SD as Determined Via DSC	Transferred Amount of Drug after 1 h (%) ± SD
Fenofibrate	40 ± 3	10 ± 0.6	88 ± 8	45.3 ± 2	90 ± 0.8
Cannabidiol	40 ± 1	9 ± 0.2	90 ± 6	46.0 ± 4	88 ± 2
Retinyl acetate	39 ± 2	10 ± 0.4	79 ± 3	43.4 ± 3	71 ± 4
Orlistat	36 ± 1 *	9 ± 0.1 *	81 ± 3 *	42.5 ± 0.4 *	36 ± 13
Lumefantrine	42 ± 3	10 ± 0.8	90 ± 7	45.1 ± 4	47 ± 6
Lumefantrine pH = 10.8	39 ± 1 *	9 *	87 ± 2 *	44.1 ± 2 *	11 ± 5

## Data Availability

The data are available upon request.
